# The Physicochemical, Textural, Microbiological and Sensory Properties of Skimmed Buffalo Milk Yoghurt with Tragacanth Gum During Storage

**DOI:** 10.17113/ftb.62.02.24.8259

**Published:** 2024-06

**Authors:** Sema Özmert Ergin, İlhan Gün, Recep Kara, Ali Soyuçok, Aslı Albayrak Karaoğlu

**Affiliations:** 1 Burdur Mehmet Akif Ersoy University, Health Sciences Faculty, Department of Nutrition and Dietetics, Degirmenler District, 15200 Yakaköy/Burdur, Turkey; 2 Burdur Mehmet Akif Ersoy University, Food Agriculture and Livestock Vocational School, Department of Food Processing, Degirmenler District, 15200 Yakaköy/Burdur, Turkey; 3 Afyon Kocatepe University, Veterinary Faculty, Department of Food Hygiene and Technology, Gazlıgöl Street, 03200 Afyonkarahisar, Turkey; 4 Burdur Mehmet Akif Ersoy University, Agriculture Livestock and Food Research Application and Research Center, Degirmenler District, 15200 Yakaköy/Burdur, Turkey

**Keywords:** buffalo milk, yoghurt, tragacanth gum, texture, microbiological properties

## Abstract

**Research background:**

In the food industry, research interest in the functional effects of natural polysaccharides from plants has increased in recent years. Tragacanth gum is used in dairy products because of its stabilising, thickening, fat-replacing and prebiotic properties. However, skimmed milk is considered a significant commercial loss in the production of buffalo clotted cream. Therefore, the aim of the present study is to investigate the potential of tragacanth gum in the production of yoghurt from buffalo milk residues with different concentrations of tragacanth gum (0.5, 1 and 1.5 g/L).

**Experimental approach:**

Skimmed buffalo milk with different concentrations of tragacanth gum was pasteurised and, after cooling at 45 °C, a starter culture was added to each sample. All samples were fermented to a pH=4.80±0.2. The gross composition, acidity, water activity, water-holding capacity, whey separation, mass fractions of organic acids and volatile aroma compounds, counts of total aerobic mesophilic bacteria, yeasts and moulds, *Lactococcus* spp. and *Lactobacillus* spp. as well as sensory and textural properties were analysed during 15 days storage.

**Results and conclusions:**

The results showed that the use of tragacanth gum increased the dry matter mass fraction, water-holding capacity and mass fraction of proteins in the samples, while whey separation decreased as the concentration of gum increased. The addition of gum improved textural properties and hardness of the yoghurt. In terms of consistency, the sample with 1 g/L tragacanth gum was the most reliable. In the control group, the total aerobic mesophilic bacteria count was highest on the first and last day of storage. According to the results of the sensory evaluation, the sample with 0.5 g/L tragacanth gum was the most favourable.

**Novelty and scientific contribution:**

Research has shown that the use of stabilisers in varying ratios improves the quality of yoghurt made from fat-free buffalo milk, which is a by-product of industrial production. So instead of ending up as industrial waste, it is recycled and its value is increased.

## INTRODUCTION

The consumption of milk and dairy products appears to play an important role in a healthy diet. Yoghurt, a probiotic food, is commonly made from many types of milk, including sheep’s, goat’s, cow’s and buffalo milk. The chemical properties of milk vary depending on the species. For example, buffalo milk has a higher concentration of fat, carbohydrates, proteins and minerals than cow’s milk, and buffalo yoghurt is widely considered to be of better nutritional quality and consistency (*1*, *2*). Consumers have turned to low-fat dietetic products in recent years in response to the obesity epidemic and associated metabolic disorders. However, some additives can be used in the manufacturing process to restore the original flavour and texture of dietary food. Gums are polysaccharides derived from both plants and animals and are widely used in the food industry. In this study, tragacanth gum, a natural gum of plant origin, is preferred because of its thickening, fat-replacing, stabilising and gelling properties in the production of yoghurt, cheese and ice cream (*3*).

Tragacanth gum is made from the sap of the *Astragalus* plant, which belongs to the family *Leguminosea*. This spiny plant grows in clumps and has white, yellow, pink or purple flowers. It grows mainly in dry and mountainous areas in Turkey, Iran, Syria and India. Tragacanth gum is obtained by extracting the sap from the stem of the plant in May-June and is used in the production of yoghurt, cheese and ice cream (*4*, *5*). Aziznia *et al*. (*6*) found that the addition of more than 0.5 g/L of tragacanth gum to fat-free yoghurt improves the structure and replaces the fat. Additionally, this process inhibits the water to crystallise and thus increases the stability and elasticity of ice cream (*7*).

Tragacanth gum, an acid-resistant edible hydrocolloid, was recognised as safe (GRAS) in 1961 (*8*). It was also included in the list of food additives (E413) by European Commission (*9*). The Turkish Food Codex Regulation on Food Additives permits the use of tragacanth gum in food products in our country (*10*).

Whey, buttermilk and skimmed milk are all dairy by-products. Therefore, in dairies, the product that remains after collecting the cream layer during the production of buffalo milk clotted cream is considered a by-product. Its evaluation is critical because a considerable amount of milk fat and protein is removed during the production of cream. Many studies have investigated the use of stabilisers in yoghurt, kefir and buttermilk. Few studies have investigated the use of skimmed milk after the production of buffalo clotted cream. Therefore, the present study aims to determine the quality characteristics of yoghurt made from skimmed buffalo milk with the addition of tragacanth gum.

## MATERIALS AND METHODS

### Materials

The Dairy Processing Facility of the Dairy Products and Technologies Application Research Centre of Mehmet Akif Ersoy University provided skimmed buffalo milk for experimental yoghurt samples from September to December 2021. Tragacanth gum was purchased locally (Sabri Güzel Salep & Tragacanth Store, Burdur, Turkey). It was collected from *Astragalus microcephalus* Willd. species grown in the Central Anatolia region (*11*).

### Yoghurt production

The buffalo milk was divided into four equal parts and labelled as samples without the addition of tragacanth gum (control sample A), and with the addition of 0.5, 1.0 and 1.5 g/L tragacanth gum (samples B, C and D, respectively). After 15–20 min of pasteurisation at (85±1) °C, the samples were cooled to the incubation temperature of (45±1) °C. Then, 4 % of each starter culture *Lactobacillus bulgaricus* and *Streptococcus thermophilus* (freeze-dried lactic culture C/LDPE 90; Igea Cultures, Termoli, Italy) were added to each experimental group and incubated until the pH decreased from 6.6±0.1 to 4.8±0.2. The coagulation process is influenced by the milk protein fraction, which is different in buffalo milk than in other animals (*12*). To investigate this, the samples were kept in the refrigerator at 4 °C for one night after the incubation was completed before being analysed on the first, seventh and fifteenth day. There were three production replicates and each sample was analysed twice in parallel.

### Physicochemical analysis

The pH of the yoghurt sample was measured using a pH metre (SevenCompact; Mettler Toledo, Greifensee, Switzerland) and the titratable acidity of the sample (TA in %) was determined according to Tekinsen *et al*. (*13*). A mass of 10 g of the yoghurt sample was mixed with 90 mL of pure water and a few drops of phenolphthalein were added to the resulting solution and titrated with 0.1 M NaOH solution. The dry mass of the samples was calculated using the gravimetric method according to AOAC method 16.032 (*14*). Approximately 2.5–3 g of the sample were weighed and dried until they reached a constant mass (3-4 h at 103-105 °C). After cooling the final weighings were made and the percentage of samples dry mass was calculated. The water activity (*a*_w_) of the samples was determined using a LabMaster Neo water activity measuring device (Novasina, Lachen, Switzerland). The water-holding capacity of the samples was determined according to the method of Sengul *et al*. (*15*). A mass of 5 g of yoghurt samples was weighed into a centrifuge tube and centrifuged at 2500×*g* and 10 °C for 30 min. After removing the supernatant, the mass of the precipitate was determined. According to the method described by Atamer and Sezgin (*16*), 5 g of yoghurt samples were weighed on the wet filter paper and kept at (4±1) °C for 2 h. The serum collected in the beaker was measured volumetrically and the amount of separated whey was calculated as mL/25 g.

The Gerber method was used to determine the fat content (%) of the samples (*17*). In addition, after calculating the total nitrogen content using the Kjeldahl method, the protein content of the samples was determined by multiplying the result with the coefficient 6.38, which represents the different typical reduced nitrogen content of proteins in food (*18*). To determine the ash content, 2–3 g of yoghurt samples were weighed and kept in a muffle furnace at 500–550 °C for 4–6 h, and after cooling the mass fraction of ash was determined.

The organic acid content of the samples was determined by high-performance liquid chromatography (HPLC) as follows (*19*): the standards for oxalic, tartaric, formic, malonic, lactic, acetic, citric, succinic and propionic acid used in this study were purchased from Sigma-Aldrich, Merck (Burlington, MA, USA). Stock solutions (in mg/L) of oxalic 100, tartaric 1000, formic 1000, malonic 1000, lactic 1000, acetic 1000, citric 100, succinic 1000, and propionic acid 100 were prepared. Samples were injected into a Shimadzu LC2040 Prominence HPLC system (Tokyo, Japan) with an LC20 AT pump and DAD detector, equiped with LC Solution computer package. The mobile phase was 10 mM NH_4_H_2_PO_4_ at a flow rate of 1 mL/min, an injection volume of 10 μL and a column temperature of 40 °C. CTO-10ASVp was used as the column oven and InertSustain C18 5 µm 250 mm×4.6 mm as the column. The volatile aroma components were analysed using the SPME-GC-MS method.

When analysing the volatile compounds, 10 μL of internal standard solution (consisting of 0.1 μL 2-methyl-3-heptanone and 6 μL 2-methyl-valeric acid in 1 mL) and 1 g NaCl were added to 5 g of the sample. The mixture was then heated at 40 °C for 20 min without fibre and again for 20 min with fibre. After a 5-minute warm-up period at 40 °C, the GC-MS column temperature was increased to 230 °C at a rate of 10 °C per minute, and the total processing time was 90 min. Helium was used as the carrier gas, and the flow rate was 1.2 mL per min. The sample was transferred to a GC-MS (QP2010; Shimadzu) equipped with a fibre and the resulting peaks were identified and calculated using the NIST library mass spectral data (*20*).

### Texture analysis

The texture profile analyser (TA.XT2; Stable Micro Systems, Caerphilly, UK) was used to examine the textural properties of yoghurt. The hardness, consistency and internal and external stickiness of the texture parameters were measured. The following parameters were used for the texture analysis: probe type: A/BE-d35, back extrusion RIG 35 mm DISC, test mode: compression, pre-test speed: 1.00 mm/s, test speed: 1.00 mm/s, post-test speed: 10.00 mm/s, distance: 30 %, strain: 70.0, trigger type auto (force): trigger force 0.049 N.

### Microbiological analysis

Under aseptic conditions, 10 g of the samples were placed in sterile stomacher bags and 90 mL of sterile peptone water (Oxoid, Thermo Fisher Scientific, Hants, UK) was added. The mixtures were then homogenised for 2 min in a stomacher (Interscience Bagmixer, St. Nom, France) and dilutions up to 10^-6^ were prepared. The prepared dilutions were plated in Petri dishes, and at the end of incubation, we only considered Petri dishes with 30-300 colonies. The total number of aerobic mesophilic bacteria was then calculated using plate count agar (PCA) (Merck, Darmstadt, Germany) (*21*). The total yeast and mould count was determined using the method proposed by the Food and Drug Administration (FDA) (*22*). For this, Rose Bengal Chloramphenicol (RBC) agar (Merck) and spread plate cultivation method were used. The microorganisms were counted after 5–7 days of incubation at 25 °C. We used MRS agar (Merck) for *Lactobacillus* spp. and M17 agar (Merck) for *Lactococcus* spp. (*23*).

### Sensory analysis

Ten panellists (three men and seven females between the ages of 26 and 54) with appropriate experience in rating the quality of yoghurt using Lawless and Heymann (*24*) method graded the samples on a hedonic scale for appearance (0–5), consistency (0–5), smell (0–5) and taste (0–5). A five-point hedonic scale was used to measure consumer acceptance as follows: 1=very dislike, 2=slightly dislike, 3=neither like nor dislike, 4=slightly like and 5=exceedingly like. Sensory evaluations were conducted in the sensory assessment room of the Dairy Products and Technologies Application Research Centre under fluorescent lighting. Each yoghurt sample was served in a plastic container containing 50 g of yoghurt at room temperature.

### Statistical analysis

The results were analysed as mean values and standard deviations using the SPSS 26.0 software (*25*). The effect of storage time and tragacanth gum concentrations was determined using analysis of variance (ANOVA). The Duncan’s multiple comparison test was then used to determine the differences between the results (p<0.05).

## RESULTS AND DISCUSSION

### Gross composition of yoghurt

An analysis prior to yoghurt production showed that the skimmed milk contained 4.78 % fat, 14.98 % total dry matter and 3.73 % protein (data not shown). The results of the physicochemical analysis of the yoghurt samples are shown in Table 1. The pH value of all samples was found to decrease with increasing storage time. Although the addition of tragacanth gum is thought to affect the change in pH ​​during incubation, the main reason for the increase in acidity during storage is the breakdown of lactose by lactic acid bacteria. While the pH value of samples B and C decreased significantly during storage (p<0.05), this decrease did not occur in sample D, which had the highest concentration of tragacanth (p>0.05).

**Table 1:** Physicochemical properties of yoghurt samples

		*t*(storage)/day		
Property	Sample	1	7	15
pH	A B C D	(5.12±0.13)^aA^ (4.91±0.04)^bA^ (4.93±0.08)^bA^ (5.0±0.14)^bA^	(4.87±0.04)^aB^ (4.73±0.06)^aB^ (4.73±0.07)^aB^ (4.8±0.2)^aA^	(4.8±0.1)^abB^ (4.60±0.02)^bC^ (4.61±0.01)^abC^ (4.8±0.2)^aA^
TA/%	A B C D	(1.2±0.1)^bB^ (1.29±0.00)^aC^ (1.30±0.01)^aB^ (1.2±0.2)^bA^	(1.3±0.1)^aA^ (1.41±0.07)^aB^ (1.43±0.09)^aA^ (1.3±0.3)^aA^	(1.3±0.23^abA^ (1.45±0.02)^aA^ (1.44±0.01)^aA^ (1.26±0.20)^bA^
*w*(total dry matter)/%	A B C D	(15.43±0.2)^aA^ (15.5±0.6)^aA^ (15.9±0.3)^aA^ (16.0±0.5)^aA^	(15.4±0.3)^aA^ (15.3±0.7)^aA^ (15.8±0.5)^aA^ (15.7±0.6)^aA^	(15.38±0.05)^bA^ (15.53±0.40)^abA^ (16.1±097)^aA^ (15.5±0.1)^abA^
*a*_w_	A B C D	(0.93±0.01)^aA^ (0.93±0.01)^aA^ (0.92±0.02)^aA^ (0.92±0.02)^aA^	(0.92±0.00)^aA^ (0.93±0.01)^aA^ (0.93±0.01)^aA^ (0.93±0.01)^aA^	(0.94±0.00)^aA^ (0.94±0.01)^aA^ (0.94±0.00)^aA^ (0.94±0.01)^aA^
WHC/%	A B C D	(61.5±4.8)^bAB^ (67.5±5.5)^aB^ (72.9±2.8)^aA^ (69.8±3.4)^aA^	(59.6±5.2)^aB^ (60.1±3.1)^aC^ (64.0±1.9)^aB^ (61.3±4.9)^aB^	(70.0±9.34^abA^ (75.4±7.2)^aA^ (78.0±9.4)^aA^ (64.4±2.7)^bB^
(*V*(whey)/*m*(sample))/(mL/25 g)	A B C D	(6.5±0.2)^aB^ (6.2±0.8)^aA^ (5.0±1.6)^bA^ (0.8±0.6)^cA^	(7.0±1.5)^aA^ (6.2±1.0)^abA^ (5.5±2.0)^bA^ (1.1±1.1)^cA^	(5.8±0.8)^aB^ (5.330.2)^aB^ (5.43±0.9^aA^ (1.6±1.6)^bA^
*w*(fat)/%	A B C D	(3.2±0.4)^aA^ (3.1±0.4)^aA^ (3.2±0.6)^aA^ (3.2±0.6)^aA^	(2.95±0.4)^aA^ (3.0±0.51)^aA^ (3.0±0.5)^aA^ (3.0±0.4)^aA^	(3.0±0.6)^aA^ (3.0±0.7)^aA^ (3.1±0.6)^aA^ (3.0±0.6)^aA^
*w*(ash)/%	A B C D	(1.04±0.02)^cA^ (1.00±0.03)^bAB^ (1.06±0.01)^abA^ (1.08±0.02)^aA^	(0.99±0.05)^bA^ (0.99±0.05)^bB^ (1.00±0.05)^bB^ (1.06±0.01) ^aA^	(1.02±0.00)^bA^ (1.06±0.00)^aA^ (1.05±0.02)^abAB^ (1.07±0.02)^aA^
*w*(protein)/%	A B C D	(5.15±0.05)^dB^ (5.28±0.03)^cA^ (5.41±0.06)^bA^ (5.64±0.05)^aA^	(5.20±0.04)^cA^ (5.25±0.02)^cB^ (5.39±0.05)^bA^ (5.61±0.04) ^aA^	(5.22±0.03)^dA^ (5.31±0.02)^cA^ (5.40±0.04)^bA^ (5.58±0.03)^aB^

Values are presented as mean±standard deviation. Mean values with different lower-case letters within a column indicate statistically significant differences between samples (p<0.05). Mean values with different capital letters within a column indicate statistically significant differences during storage (p<0.05). A=control sample, B, C and D=sample with added 0.5, 1.0 and 1.5 g tragacanth gum per L of milk, respectively. TA=titratable acidity, *a_w_*=water activity, WHC=water-holding capacity

As buffalo milk has a high protein content, the development of acidity of the buffalo milk yoghurt was lower than that of cow’s milk yoghurt. The pH value of skimmed buffalo milk used in production was 6.55. In terms of product quality, we chose a pH=4.80 rather than 4.6; the values on the first day of storage were therefore considered more suitable for product structure and coagulation quality. It has been shown that increasing the α_s1_-casein content of the protein fractions slows the onset of coagulation while recducing the pH, coagulation time and curd firming time (*12*). Furthermore, it is assumed that the higher casein content, higher concentration of inorganic phosphate and the presence of organic compounds with acid-base properties in buffalo milk are responsible for the higher buffering capacity of the milk (*26*).

Therefore, the coagulation properties of the samples are likely due to the differences in the ratio of protein fractions in buffalo milk. Bonfatti *et al*. (*27*) state that α(S1)-, α(S2)-, βγ- and κ-casein ratio of buffalo milk were 32.2, 15.8, 36.5 and 15.5 %. The titratable acidity of the samples also increased with storage. At the end of the storage, sample B, containing 0.5 g tragacanth gum, had the highest titratable acidity. According to Han *et al*. (*28*), the initial pH of low-fat buffalo yoghurt was 4.34, but decreased to 4.05 after ten weeks of storage. In the same study, the authors reported an increase in acidity due to continuous lactic acid fermentation during storage. Furthermore, titratable acidity increased until the sixth week, but there was no significant change in acidity between the sixth and tenth week of storage. In another study, it was found that the addition of 0.25 g tragacanth gum to cow’s milk yoghurt did not result in a significant change in acidity compared to the control sample. However, the authors emphasised that the acidity increased with the increasing amounts of added gum (*6*). The use of tragacanth gum contributes not only to the structure of the yoghurt but also to the dry matter mass fraction. Although different types of milk are used, the average dry matter mass fraction of yoghurt is often between 14 and 20 % (*29*). Nahar *et al*. (*30*) found that buffalo yoghurt had the highest dry matter mass fraction (16.86 %) among those made from cow, buffalo and goat milk. Erkaya and Sengul (*31*) determined the dry matter mass fraction of buffalo yoghurt to be 17.87 %. Another study on low-fat buffalo yoghurt showes a mass fraction of 11.60 % (*24*). Unal *et al*. (*32*) investigated the addition of locust bean gum to low-fat yoghurt. They found that dry matter mass fraction increased and the viscosity in the yoghurt samples decreased with the increase in gum content. The appropriate mass fractions of gum in milk powder are 0.02 g/100 g and dry matter 14 %. The yoghurt samples had water activity values ranging from 0.92 to 0.94 (Table 1). Furthermore, the values on the first and seventh day of storage differed insignificantly from those on the fifteenth day (p>0.05). Tayar *et al.* (*33*) determined the water activity values of yoghurt samples containing stabilisers in different ratios of 0.85-0.95 and found that the water activity decreased with increasing stabiliser ratio.

The water-holding capacity of samples with different ratios of tragacanth gum was found to be substantially affected by both the rate of gum addition and the storage time (p<0.05). On the first day of storage, the control sample had the lowest water-holding capacity, but this increased depending on the concentration of gum in samples B and C. The product with the highest water-holding capacity after 15 days of storage was sample C (78.02 %). In an earlier study, the water-holding capacity of yoghurt made from 4 % fat buffalo milk was estimated to be 86.8 % (*34*). Dusunen (*35*) estimated the water-holding capacity of buffalo yoghurt marketed in Tekirdag province, Turkey, during the winter months to be 93.15−95.51 %. However, in the samples taken in the spring months, it was determined to be 88.58–90.78 %. The separation of the whey trapped in the protein network from the gel-like structure appears to be a fundamental structural flaw in yoghurt. Many approaches are taken in the current dairy industry to avoid whey separation, such as the use of stabilisers, increasing the dry matter of the milk or denaturing whey protein by extended heat treatment at high temperatures (*36*). Whey separation was found to be considerably lower in the samples when compared on the first day, especially in sample D with the addition of 1.5 g/L tragacanth gum (p<0.05). Atasever (*37*) investigated the effect of stabilisers on yoghurt and found that whey separation was 5.0–6.27 mL/25 g in agar samples, 4.10–5.63 mL/25 g in gelatine samples and 3.61–6.10 mL/25 g in Na-alginate samples. The nutrient composition of buffalo milk contributes significantly to the nutritional quality of buffalo yoghurt. The fat mass fraction of the samples in this study was between 2.95 and 3.25 %, although there were no significant changes in fat content between the samples during storage time (p>0.05). Samples C and D had the highest fat mass fraction (3.2±0.6) % on the first day of storage. The mineral content of yoghurt is related to its ash mass fraction. The ash mass fraction of the samples ranged between 0.99 and 1.08 %. Furthermore, sample D had the highest ash mass fraction. According to Dusunen (*35*), the fat and ash mass fractions of buffalo yoghurt were between 6.72–7.13 % and 0.87–0.93 %, respectively. The ash mass fraction and hence mineral content of the samples from skimmed buffalo milk were higher in this study. Another study investigated yoghurt made from skimmed cow’s milk and tragacanth gum. The results show that low-fat yoghurt samples had a higher ash and protein mass fraction. It was also found that the sample with the highest ash mass fraction was the one containing 0.75 g (0.99 %) gum (*6*). Madadlou *et al*. (*38*) found that the reduction in fat content in milk results in a proportional increase in the ratio of water to protein. This increase in water and protein ratio subsequently leads to an increase in the water-soluble mineral matter, which affects the ash content. We found that the samples in the control group had a significantly lower protein mass fraction than the other groups. The higher protein mass fraction in the samples can thus be at least partly attributed to the increased concentrations of tragacanth gum. The protein mass fraction in all samples used in this analysis was measured between 5.15 and 5.64 %. Although the yoghurt samples were made from milk residues after cream production, they had a high protein content. It is likely that our samples retained their nutrient content because the milk protein is concentrated in the liquid that is separated from the cream. Sahsi (*39*) found that the protein mass fraction of buffalo yoghurt ranged from 5.08 to 5.22 %, based on an analysis of the effects of using frozen buffalo milk in yoghurt preparation. In addition, Erkaya and Sengul (*31*) and Nahar *et al*. (*30*) found that buffalo yoghurt contained 4.67 and 4.25 % protein, respectively. Another study found that although buffalo yoghurt had a total protein mass fraction of 4.97 %, it decreased to 3.56 % when corn and soy milk were used in the preparation (*40*).

### Textural properties of yoghurt

The textural analysis of yoghurt samples determined values for hardness, consistency and internal and external stickiness, which are shown in Table 2. The acidity of the milk, the amount of dry matter in the milk and the protein content play an important role in determining the settling properties of yoghurt. Buffalo milk, which contains more fat than cow’s milk, is not used for drinking but is processed into cream, cheese and yoghurt (*41*). Yoghurt made from buffalo milk has a solid texture due to the high dry matter content of the milk (*42*). We found that the hardness of the samples increased when tragacanth gum was added compared to the hardness of the control sample. Besides, the longer these samples were kept in storage, the harder they were (p<0.05). Sample C, with added 1.0 g/L of gum, had the highest hardness ratings on both the seventh and fifteenth day of storage. However, the firmness of the yoghurt decreased when more than 1.0 g/L gum was added. The fat-replacing properties of tragacanth gum in fat-free yoghurt samples were investigated by Aziznia *et al*. (*6*). Their results showed that adding more than 0.5 g/L of gum to yoghurt did not significantly change its consistency. The sample to which 0.25 g/L of tragacanth gum was added was the hardest one they tested. Our results also showed that the consistency of samples B and C improved after tragacanth gum was added. On the fifteenth day of storage, sample C was the most consistent sample. However, the uniformity of the samples was affected by higher concentrations of gum. In addition, the internal stickiness values were shown to be maximal for all samples on the seventh day of storage. The external stickiness of the samples was affected by the addition of gum; in control samples it was significantly lower than in other samples (p<0.05). Huang *et al*. (*43*) investigated the effects of polydextrose (a water-soluble dietary fibre) on fat-replacing function and organoleptic/textural structure of fat-free buffalo yoghurt. The authors found that the hardness, stickiness and cohesiveness values of the samples produced with 1.5, 3 and 5 % polydextrose were all higher than in the control sample. The literature also shows that xanthan gum and locust bean gum improve the consistency and hardness of yoghurt (*44*).

**Table 2:** Textural properties of yoghurt samples

		*t*(storage)/day		
Property	Sample	1	7	15
Hardness/N	A B C D	(174±2)^cB^ (500±5)^aC^ (414±4)^bC^ (506±5)^aB^	(147±1)^cC^ (550±5)^bB^ (622±6)^aB^ (539±5)^Ba^	(199±2)^dA^ (641±6)^bA^ (776±8)^aA^ (553±5)^cA^
Consistency/(N∙s)	A B C D	(12069±120)^bC^ (14876±147)^aB^ (14709±146)^aC^ (1974±20)^cA^	(14116±140)^cB^ (15395±152)^bB^ (24234±240)^aB^ (1176±12)^dB^	(16292±161)^cA^ (19309±191)^bA^ (25293±250)^aA^ (882±9)^dC^
Internal stickiness/N	A B C D	(-184±2)^bA^ (-115±1)^aA^ (-552±5)^cA^ (-850±8)^dA^	(-422±4)^aC^ (-1125±11)^bC^ (-1742±17)^cC^ (-1164±12)^bC^	(-325±3)^aB^ (-960±10)^bB^ (-1242±12)^cB^ (-953±9)^bB^
External stickiness/(g∙s)	A B C D	(-86±1)^aA^ (-183±2)^bA^ (-255±3)^cA^ (-414±4)^dA^	(-132±1)^aB^ (-427±4)^cB^ (-582±6)^dC^ (-406±4)^bA^	(-161±2)^aC^ (-434±4)^bB^ (-459±5)^cB^ (-441±4)^bB^

Values are presented as mean±standard deviation. Mean values with different lower-case letters within a column indicate statistically significant difference between samples (p<0.05). Mean values with different capital letters within a column indicate statistically significant difference during storage (p<0.05). A=control sample, B, C and D=sample with added 0.5, 1.0 and 1.5 g tragacanth gum per L of milk, respectively

### Mass fractions of organic acids in yoghurt

The characteristic flavour and aroma of yoghurt are the result of the fermentation of milk, which is induced by the addition of starter cultures (*45*). Organic acids such as lactic, acetic, formic, succinic and citric acids are a by-product of the fermentation process. They also promote the synthesis of nucleic acids and inhibit microbial growth. The organic acid content and probiotic function of yoghurt give it a prominent place in nutrition (*46*).

Lactic acid was the most abundant organic acid in the samples (Table 3). On the first day of storage, the control sample had a higer lactic acid mass fraction than the sample with the added tragacanth gum. However, we found that after a decrease on the seventh day (p<0.05), the lactic acid mass fraction in samples B and C increased significantly again. This was in contrast to the trend observed in the control sample, where the lactic acid mass fraction decreased with increasing storage time. The mass fractions of acetic, formic, succinic and oxalic acids were the highest in sample A on the first day of storage. The content of lactic acid in the samples is much higher than malonic acid. Sample B had the highest mass fraction of malonic acid with 1805.66 µg/kg, followed by sample A with 866.35 µg/kg and sample D with 697.51 µg/kg. In addition, malonic acid content decreased significantly during storage.

**Table 3:** Mass fractions of organic acids in yoghurt samples

														*t*(storage)/day											
	1	7	15	1	7		15		1	7		15		1	7	15	1	7	15	1	7	15	1	7	15
Sample														*w*(acid)/(mg/kg)											
	Lactic			Tartaric					Acetic					Formic			Citric			Succinic			Oxalic		
A	(17299±69)^aA^	(17355±69)^aA^	(14742±6)^bA^	(3300±13)^aA^		(2882±12)^bA^		(2409±10)^cA^	(2355±9)^aA^		(1078±4)^bC^		(945±4)^cC^	(1696±7)^aA^	(1386±6)^bA^	(836±3)^cC^	(604±2)^cD^	(1145±5)^bD^	(1890±8)^aB^	(1907±8)^aA^	(1481±6)^aB^	(660±3)^bC^	(245±1)^aA^	(220±1)^aB^	(214±1)^aC^
B	(16864±67)^aB^	(13400±53)^cC^	(13743±6)^bB^	(2464±10)^aB^		(2216±9)^bC^		(2170±9)^cC^	(2087±8)^aB^		(854±3)^bD^		(842±3)^bD^	(1358±5)^aB^	(1081±4)^bB^	(770±3)^cD^	(1633±7)^cC^	(1992±8)^bA^	(2045±8)^aA^	(869±3)^cA^	(767±3)^cB^	(511±2)^dC^	(138±1)^dC^	(183±1)^cA^	(179±1)^bB^
C	(14050±56)^aC^	(11829±47)^cD^	(12469±50)^bC^	(2391±10)^aC^		(2098±8)^bD^		(1574±6)^cD^	(933±4)^cC^		(1118±5)^bB^		(1252±5)^aB^	(999±4)^aC^	(936±4)^bC^	(920±4)^bB^	(1846±7)^aA^	(1599±6)^bC^	(1152±5)^cD^	(427±2)^dC^	(683±3)^dB^	(1066±4)^aA^	(175±1)^cB^	(194±1)^bA^	(246±1)^cC^
D	(12826±51)^bD^	(13726±55)^aB^	(13909±55)^aB^	(2362±9)^bC^		(2554±10)^aB^		(2254±9)^cB^	(866±4)^cD^		(2308±9)^bA^		(2350±9)^aA^	(879±4)^bD^	(880±4)^bD^	(1089±4)^aA^	(1706±7)^aB^	(1682±7)^aB^	(1583±6)^bC^	(1092±4)^bA^	(905±4)^bB^	(560±2)^cC^	(210±1)^bC^	(219±1)^aA^	(215±1)^aB^

Values are presented as mean±standard deviation. Mean values marked with different lowercase letters within a column indicate statistically significant difference between samples (p<0.05). Mean values marked with different capital letters within a column indicate statistically significant difference during storage (p<0.05). A=control sample, B, C and D=sample with added 0.5, 1.0 and 1.5 g tragacanth gum per L of milk, respectively

Yoghurt flavour highly depends on succinic acid, but we found that it decreased in all samples except sample C. Initial mass fractions of 1907 mg/kg in sample A, 869 mg/kg in sample B and 1092 mg/kg in sample D decreased to 660, 511 and 560 mg/kg, respectively, by the end of storage. In sample C, an increase from 427 mg/kg to 1066 mg/kg was detected. The oxalic acid mass fraction was consistently the lowest among the measured organic acids. The oxalic acid mass fraction of sample A decreased during storage from 245 to 214 mg/kg, while samples B, C and D showed an increase from 138 to 179 mg/kg, from 175 to 246 mg/kg and from 210 to 215 mg/kg, respectively. Buffalo yoghurt is characterised by the presence of lactic and citric acids in higher amounts. Nguyen *et al*. (*47*) reported that the amounts of lactic, acetic and pyruvic acids increased during storage, while the contents of the other organic acids remained constant.

### Volatile aroma compounds in yoghurt

The essential flavour of yoghurt is attributed to the presence of non-volatile acids (such as lactic, pyruvic, oxalic and succinic acids), volatile compounds (including butyric, acetic and propionic acids) and carbonyl compounds (such as acetaldehyde, diacetyl, acetone and acetoin). These compounds are synthesised by the activities of starter cultures *Lactobacillus delbrueckeii* ssp. *bulgaricus* and *Streptococcus thermophilus*, which are suitable for symbiotic growth in yoghurt production (*45*). It has also been claimed by several scientists that acetaldehyde, ethanol, acetone, diacetyl and 2-butanone play an essential role in shaping the sensory properties of yoghurt (*48*).

The mass fractions of volatile chemicals determined by the gas chromatography-mass spectrometry (GC-MS) and solid-phase microextraction (SPME) method are shown in Table 4. The results of the study showed the presence of 32 volatile chemicals at different mass fractions in our samples over a 15-day storage period. Among the identified compounds, ethanol, diacetyl, acetoin, acetic acid, 1-hexanol-2-ethyl, 6-methyl-1-octanol, butanoic acid and hexanoic acid were found in large quantities. The diacetyl mass fraction of the samples was in a range of 2-71 mg/kg during the initial seven-day storage period, but it was not detectable on the fifteenth day of storage. On the first day, samples B and C had a significantly higher diacetyl mass fraction than the control sample (p<0.05). However, after seven days of storage, the control sample exceeded the other samples with a diacetyl mass fraction of 71 mg/kg (p<0.05). Acetaldehyde was detected in all yoghurt samples on the first day, but the trend was not the same on the following days. The volatile component acetoin showed statistically significant (p<0.05) changes in all samples during storage. It was also found that the acetoin mass fraction in samples A and D was similar on the first day of storage. However, samples B and C had approximately three and six times higher acetoin content, respectively. On the last day of storage, sample C had the highest mass fraction of acetoin, while the subsequent samples B, D, and A showed progressively lower values. The presence of acetic acid was much more pronounced than that of other volatile compounds. Another significant volatile compound identified in this investigation was 1-hexanol-2-ethyl. With the exception of sample C on the fifteenth day of storage, the highest value was detected in sample B on the fifteenth day and in sample A on the seventh day (p<0.05). The mass fractions of 6-methyl-1-octanal, butanoic acid and hexanoic acid showed remarkable alterations throughout the storage period. The compound 6-methyl-1-octanol was not observed in sample A on both the first and fifteenth day. The mass fractions of 6-methyl-1-octanol, butanoic acid and hexanoic acid changed substantially during storage. On day 1 and day 15, 6-methyl-1-octanol was not detected in sample A, but on day 1, it was found in sample B at a mass fraction of 92 mg/kg and on day 15, it was found in sample C at 275 mg/kg. Sample B had the highest butanoic acid content on day 1 (302 mg/kg), while sample A had the highest butanoic acid content on days 7 and 15 at 825 and 607 mg/kg, respectively. It was also found that there was a statistically significant difference (p<0.05) in the butanoic acid mass fraction in the samples after storage. Erkaya and Sengul (*30*) analysed volatile compounds in the yoghurt made from cow’s, sheep’s, goat’s, and buffalo milk. According to their results, buffalo milk contained significantly higher amounts of acetaldehyde and caproic acid than the other tested milk samples. However, ethyl acetate was detected in higher contents in the cow’s and goat’s milk samples than in the buffalo yoghurt. According to Emirmustafaoglu *et al*. (*49*), the most abundant volatile chemicals in the yoghurt samples were acetaldehyde (8.93 mg/kg), ethanol (114.93 mg/kg), diacetyl (0.95 mg/kg), acetoin (24.44 mg/kg) and acetone (0.59 mg/kg). According to Guzeler *et al*. (*50*), acetaldehyde plays a crucial role in the flavour profile of yoghurt. However, in the case of buffalo yoghurt, it is not considered a prominent flavour compound due to its subsequent conversion into alcohol. Nevertheless, the samples were found to have increased mass fractions of acetic acid (35.249 %), butanoic acid (4.742 %) and hexanoic acid (3.047 %) compared to other acid compounds. The samples showed high mass fractions of isoamyl alcohol (5.349 %), 2-methyl-2-pentanol (2.629 %), acetoin (20.731 %) and vinyl acetate (4.224 %). Buffalo yoghurt samples with the addition of 1 % whey protein concentrate (WPC) and 1 % calcium caseinate had acetic acid mass fractions of 6.22–16.23 mg/100 g in the control sample, 7.99-20.18 mg/100 g in the WPC sample and 7.30-18.10 mg/100 g in the calcium caseinate sample (*51*). The butanoic acid content in the samples ranged from 20.89 to 20.94 mg/100 g before it was undetectable on the 21st day of storage, according to the authors.

**Table 4:** Volatile compound content in yoghurt samples

		Sample												
		A			B			C			D			
RT	Volatile compound	*t*(storage)/day												
		1	7	15	1	7	15	1	7	15	1	7		15
		*w*(volatile compound)/(mg/kg)												
3930	Carbon dioxide				(423±12)					(183±2)				
5960	Ethyl acetate	(53.0±0.2)^aA^			(25.0±0.3)^bA^			(21.0±0.2)^bA^			(19.0±0.8)^cA^			
6605	2-Butanol	(12.00±0.01)^aA^			(9.0±0.2)^bA^									
6720	Ethanol	(35.0±0.5)^bC^	(45.0±0.6)^bB^	(119±14)^aA^	(55.0±0.6)^aA^	(57.0±0.2)^bA^	(43.0±0.3)^bB^	(17.0±0.1)^cB^	(36.0±0.3)^cA^	(61.0±0.9)^cAA^	(29.0±1.3)^bC^	(350±13)^aA^		(88±1)^bB^
8574	Diacetyl	(11.0±0.1)^bB^	(71.0±0.4)^aA^		(27.0±0.3)^aA^	(10.0±0.2)^bB^		(23.0±0.3)^aA^	(3.0±0.1)^cB^		(19.0±0.3)^bA^	(2.0±0.5)^cB^		
8736	Toluene	(18.0±0.6)^aB^			(114±5)^aA^					(94±2)^aA^				
9695	4-Octanone					(14.0±0.1)								
10064	1-Propanol-2-methyl	(30.0±0.3)												
11479	Acetaldehyde	(2.0±0.2)^bA^			(2.0±0.4)^bA^			(3.0±0.1)^aA^			(2.0±0.1)^bA^			
12438	2-Hexanone-4-methyl				(10.0±0.5)^aA^									
13050	Formic acid			(510±31)^a^			(453±22)^a^			(45.0±0.4)^b^	(792±18)			
13088	1-Butanol-3-methyl	(284±19)^aA^	(95±3)^bA^	(55±3)^cA^	(14.0±0.6)^bA^									
15897	Acetoin	(25.0±0.6)^cC^	(160±14)^aA^	(92±4)^bB^	(162±18)^aA^	(37±2)^bC^	(134±3)^aB^	(81.0±0.7)^bB^	(39.0±0.3)^b^ ^C^	(164±24)^aA^	(25.0±0.8)^cB^	(35.0±0.9)^cB^		(101±15)^bA4^
16785	Oxalic acid				(494±36)^bA^				(331±11)^aA^	(537±32)^bA^	(1229±45)^aA^			
18244	1-Pentene-2-methyl	(17.0±0.2)^bA^					(63.0±0.5)^aA^	(11.0±0.2)^bA^			(56.0±0.7)^aA^			(9.0±0.5)^aB^
18249	1-Hexanol		(17.0±0.4)^aA^	(12.0±0.2)^aA^	(9.0±0.3)^bB^					(98±2)^aA^	(14.0±0.3)^aA^	(11.0±0.2)^aA^		
19451	Benzoctamine	(4.0±0.1)^aA^												
22877	Acetic acid	(2033±42)^bB^	(8502±35)^aA^	(2672±52)^bB^	(4954±54)^aA^	(973±12)^cC^	(1268±23)^cB^	(921±32)^cB^	(952±19)^cB^	(3100±21)^aA^	(1907±34)^bB^	(5131±42)^bA^		(1153±14)^cB^
23406	Furaldehyde			(13.1±0.2)^aA^							(9.0±0.6)^aA^			
24386	1-Hexanol-2-ethyl	(21.0±0.6)^bB^	(55.0±0.9)^aA^	(52.0±1.4)^bA^	(31.0±0.3)^aB^	(20.0±0.9)^bC^	(206.0±2.8)^aA^	(10.0±0.5)^dA^	(6±1)^cB^		(14.0±0.6)^cA^	(5.0±0.1)^cC^		(11.0±0.3)^cA^
25031	2-Mercapto-4-phenylthiozole			(22.0±0.3)										
25604	4-Hydroxymandelic acid		(91.0±0.4)											
25929	Benzaldehyde	(8.0±0.2)												
26887	2-3-Butanediol	(37.0±1.1)										(120±4)		
27923	1-Butanol-2-ethyl		(12.0±0.5)							(90±3)				
29757	1-Octanol-2-methyl		(7.0±0.3)^aB^	(12.0±0.2)^bA^	(15.0±0.5)^A^	(7.0±0.1)^aB^				(43±2)^a^				
31195	2-Hexanal					(14.0±0.2)								
31366	6-Methyl-1-octanol		(52.0±0.5)^a^		(92.0±0.3)^aB^	(41.0±0.5)^aC^	(322±6)^aA^	(32.0±0.9)^bC^	(9.0±0.6)^cA^	(275.0±4.8)^aA^	(43.0±0.7)^bA^	(12.0±0.4)^bC^	(26±1)^bB^	
31663	Butanoic acid	(202±27)^bC^	(825±52)^aA^	(607±41)^aB^	(302±25)^aB^	(34±2)^dC^	(427±18)^bA^	(301±13)^aB^	(233±17)^bC^	(590±36)^aA^	(122±12)^cB^	(113±25)^cB^	(296±19)^cA^	
32980	1-Nonanol		(10.0±0.2)		(37.0±0.8)^aA^		(2.0±0.1)^bB^			(89.0±0.4)^b^	(11.0±0.3)^b^			
32985	2-Furanmethanol			(92.0±0.7)^a^		(16.0±0.4)^b^				(77.0±0.3)^a^		(12.0±0.2)^bB^	(29.0±0.6)^bA^	
42697	Hexanoic acid		(704±24)^aA^	(398±15)^bB^	(755±13)^aA^	(274±17)^cB^	(337±12)^bB^	(265±15)^cC^	(388±21)^bB^	(561±26)^aA^	(569±20)^bA^	(422±18)^bB^	(342±23)^bB^	

Values are presented as mean±standard deviation. Mean values marked with different lowercase letters within a column indicate statistically significant difference between samples (p<0.05). Mean values marked with different capital letters within a column indicate statistically significant difference during storage (p<0.05). A=control sample, B, C and D=sample with added 0.5, 1.0 and 1.5 g tragacanth gum per L of milk, respectively

### Microbiological properties of yoghurt

The data in Table 5 show the number of total aerobic mesophilic bacteria (TAMB), yeasts/moulds, *Lactobacillus* spp. and *Lactococcus* spp. Although sample B had the lowest TAMB count on the first day of storage, the control sample had the highest number of bacteria on both the first and last day of storage. Additionally, a notable decrease in yeast/mould counts was observed in all samples during the later stages of storage (p<0.05). Sample B, which contained 0.5 g of tragacanth gum, had the lowest yeast/molud count on the fifteenth day. The yeast/mould count was between 2.8 and 7.05 log CFU/g in all samples. The microbiological quality of buffalo yoghurt was investigated in one study and the TAMB, yeast and mould counts of the yoghurt samples ranged 5.40–9.80, 4.00–7.50 and 3.98–6.48 log CFU/g, respectively (*2*).

**Table 5:** Microbial counts in yoghurt samples

		*N*(microorganism)/(log CFU/g)		
Microorganism	Sample		*t*(storage)/day	
		1	*7*	15
TAMB	A B C D	(7.8±0.4)^aAB^ (6.95±0.07)^bC^ (7.50±0.06)^aC^ (7.81±0.08)^aAB^	(7.59±0.02)^dB^ (8.57±0.05)^aA^ (8.35±0.04)^bA^ (8.01±0.02)^cA^	(8.21±0.07)^aA^ (7.51±0.09)^cB^ (7.90±0.02)^bB^ (7.5±0.2)^cB^
Yeast and mould	A B C D	(6.86±0.01)^abA^ (7.05±0.11)^aA^ (6.4±0.4)^bA^ (6.99±0.02)^aA^	(6.73±0.01)^abA^ (6.61±0.05)^bB^ (6.7±0.1)^bA^ (6.87±0.01)^aA^	(4.1±0.1)^bB^ (2.8±0.1)^dC^ (3.29±0.02)^cB^ (4.40±0.01)^aB^
*Lactobacillus* spp.	A B C D	(7.5±0.3)^abB^ (7.22±0.06)^bcA^ (7.75±0.03)^aA^ (7.01±0.05)^bB^	(8.48±0.01)^aA^ (7.26±0.06)^dA^ (7.42±0.08)^cB^ (7.81±0.03)^bA^	(7.21±0.07)^aB^ (6.51±0.09)^cB^ (6.90±0.02)^bC^ (6.5±0.2)^cC^
*Lactococcus* spp.	A B C D	(8.2±0.4)^aB^ (8.0±0.6)^aB^ (8.1±0.2)^aB^ (7.9±0.2)^aB^	(9.43±0.08)^abA^ (9.09±0.07)^bA^ (9.69±0.02)^aA^ (9.2±0.5)^abA^	(6.8±0.2)^bC^ (6.1±0.1)^cC^ (6.90±0.04)^abC^ (7.06±0.05)^aC^

Values are presented as mean±standard deviation. Mean values marked with different lowercase letters within a column indicate statistically significant difference between samples (p<0.05). Mean values marked with different capital letters within a column indicate statistically significant difference during storage (p<0.05). A=control sample, B, C and D=sample with added 0.5, 1.0 and 1.5 g tragacanth gum per L of milk, respectively; TAMB=total aerobic mesophilic bacteria

The production of lactic acid by lactic acid bacteria during the process of milk fermentation is widely recognised as a key factor contributing to the characteristic flavour and aroma properties of yoghurt. Lactic acid bacteria also play a crucial role in protecting against spoilage by inhibiting the proliferation of pathogenic microbes. They are known for their antibacterial, anticancer and immune system-enhancing properties (*52*). Development of *Lactobacillus* spp. colonies on MRS agar was similar in all yoghurt samples on the first day of production. However, it was observed that the colony count was higher in the control sample on the seventh and fifteenth day. The number of *Lactococcus* spp. grown on M17 agar increased in all samples on the seventh day, followed by a subsequent decrease on the fifteenth day. They reached the highest level in sample C on the seventh day with a count of 9.69 log CFU/g. Therefore, it was concluded that the addition of tragacanth gum did not have a negative effect on the fermentation process of yoghurt made from skimmed buffalo milk.

### Sensory properties

We found deficiencies in the increased addition of the tragacanth gum to buffalo milk yoghurt (Table 6), but in a sensory evaluation that considered the appearance, consistency, smell and taste of the product, we found that the addition of tragacanth gum of 0.5 g/L improved the yoghurt quality. The panellists gave the lowest score to sample D, with 1.5 g/L tragacanth gum, due to the more gelatinous structure and insipid flavour of the sample. In terms of consistency, the effect of using 0.5 g/L gum proved to be more significant (p<0.05). The control sample and sample B received higher scores for smell, but the preference rate decreased as more gum was added. While the effect of low concentrations of tragacanth gum on taste and odour was not statistically significant, the increase in its addition had a negative effect on the panellists' preference rating. During 15 days of storage, sample D was not able to develop a slightly acidic flavour, which is a preferred flavour for yoghurt. This could be because the flavour of the gum masked the taste of the sample.

**Table 6:** Sensory evaluation scores of yoghurt samples

	Appearance			Consistency			Smell			Taste		
Sample						*t*(storage)/day						
	1	7	15	1	7	15	1	7	15	1	7	15
A	(3.83±0.01)^bB^	(4.00±0.02)^aAB^	(4.33±0.02)^bA^	(4.83±0.03)^abA^	(4.00±0.02)^bC^	(4.17±0.02)^bB^	(4.67±0.01)^bA^	(4.50±0.03)^bB^	(4.33±0.02)^aC^	(3.67±0.04)^bB^	(4.00±0.01)^bAB^	(4.17±0.02)^bA^
B	(4.17±0.03)^aC^	(4.33±0.02)^aB^	(5.00±0.02)^aA^	(5.00±0.01)^aA^	(4.50±0.01)^aB^	(4.67±0.01)^aB^	(4.83±0.02)^aA^	(4.67±0.01)^aB^	(4.17±0.02)^bC^	(4.17±0.01)^aB^	(4.50±0.02)^aA^	(4.50±0.02)^aA^
C	(3.33±0.02)^cB^	(3.50±0.01)^bB^	(4.00±0.01)^bA^	(4.17±0.02)^bA^	(4.00±0.02)^bB^	(4.17±0.02)^bA^	(4.17±0.01)^cC^	(4.67±0.02)^aA^	(4.33±0.01)^aB^	(3.83±0.02)^bC^	(4.50±0.03)^aA^	(4.33±0.00)^abB^
D	(3.17±0.01)^cA^	(2.50±0.03)^cB^	(3.00±0.03)^cA^	(2.67±0.01)^cA^	(2.17±0.02)^cC^	(2.50±0.03)^cB^	(3.50±0.02)^dC^	(4.33±0.01)^cA^	(4.00±0.02)^cB^	(2.17±0.03)^cC^	(2.50±0.02)^cA^	(2.33±0.03)^cB^

Values are presented as mean±standard deviation. Mean values marked with different lower-case letters within a column show statistically different between samples (p<0.05). Mean values marked with different capital letters within a column are statistically different during storage (p<0.05). A=control sample, B, C and D=0.5. 1.0 and 1.5 g TG per L of milk, respectively

Neto *et al*. (*53*) tested buffalo milk yoghurts with 5, 3 and 6 % fat and found that consumers preferred the higher fat versions. According to Erkaya and Sengul (*31*), acetaldehyde content is an important factor in the distinctive flavour and aroma of buffalo yoghurt. Nahar *et al*. (*30*) reported that despite the higher nutritional value of buffalo yoghurt, the panellists in the study did not favour buffalo yoghurt much.

## CONCLUSIONS

The aim of this study was to investigate the physicochemical, textural, microbiological and sensory properties of yoghurt made from skimmed buffalo milk with the addition of different concentrations of tragacanth gum. The results showed that the use of tragacanth gum had a positive effect on the overall quality of the yoghurt. It was also found that the optimal concentration of tragacanth gum in the yoghurt production is 1 g/L. This finding is of great importance for the overall quality of the final product. In general, it is believed that tragacanth gum can be used in various dairy products and that its ability to replace fat can be taken into account in the development of dietary products.
